# Physiological Fontan Procedure

**DOI:** 10.3389/fped.2019.00196

**Published:** 2019-05-24

**Authors:** Antonio F. Corno, Matt J. Owen, Andrea Cangiani, Edward J. C. Hall, Aldo Rona

**Affiliations:** ^1^University of Leicester, Leicester, United Kingdom; ^2^School of Mathematical Sciences, University of Nottingham, Nottingham, United Kingdom; ^3^Department of Engineering, University of Leicester, Leicester, United Kingdom

**Keywords:** congenital heart defects, congenital heart surgery, Fontan procedure, flow modeling, computational fluid dynamics, performance prediction, physiological design, univentricular heart

## Abstract

**Objective:** The conventional Fontan circulation deviates the superior vena cava (SVC = 1/3 of the systemic venous return) toward the right lung (3/5 of total lung volume) and the inferior vena cava (IVC = 2/3 of the systemic venous return) toward the left lung (2/5 of total lung volume). A “physiological” Fontan deviating the SVC toward the left lung and the IVC toward the right lung was compared with the conventional setting by computational fluid dynamics, studying whether this setting achieves a more favorable hemodynamics than the conventional Fontan circulation.

**Materials and Methods:** An *in-silico* 3D parametric model of the Fontan procedure was developed using idealized vascular geometries with invariant sizes of SVC, IVC, right pulmonary artery (RPA), and left pulmonary artery (LPA), steady inflow velocities at IVC and SVC, and constant equal outflow pressures at RPA and LPA. These parameters were set to perform finite-volume incompressible steady flow simulations, assuming a single-phase, Newtonian, isothermal, laminar blood flow. Numerically converged finite-volume mass and momentum flow balances determined the inlet pressures and the outflow rates. Numerical closed-path integration of energy fluxes across domain boundaries determined the flow energy loss rate through the Fontan circulation. The comparison evaluated: (1) mean IVC pressure; (2) energy loss rate; (3) kinetic energy maximum value throughout the domain volume.

**Results:** The comparison of the physiological vs. conventional Fontan provided these results: (1) mean IVC pressure 13.9 vs. 14.1 mmHg (= 0.2 mmHg reduction); (2) energy loss rate 5.55 vs. 6.61 mW (= 16% reduction); (3) maximum kinetic energy 283 vs. 396 J/m^3^ (= 29% reduction).

**Conclusions:** A more physiological flow distribution is accompanied by a reduction of mean IVC pressure and by substantial reductions of energy loss rate and of peak kinetic energy. The potential clinical impact of these hemodynamic changes in reducing the incidence and severity of the adverse long-term effects of the Fontan circulation, in particular liver failure and protein-losing enteropathy, still remains to be assessed and will be the subject of future work.

## Introduction

The principle of the Fontan circulation, successfully introduced by Frances Fontan for a patient with tricuspid atresia (1) in the early seventies, has been since applied to a huge variety of congenital heart defects, with various morphologies. All these complex congenital heart defects share the same characteristic of having “functionally” univentricular hearts, because of the presence of only one ventricle morphologically and/or functionally adequate to support the systemic circulation, pumping the oxygenated blood into the aorta (2–7). In the Fontan circulation, the low oxygen saturation blood returning from the superior vena cava (SVC) and from the inferior vena cava (IVC) is deviated directly through the lungs by various types of surgical connections with the pulmonary arteries (8).

A preparation is generally required in the neonatal period to either increase (9–11) or decrease (12–17) the pulmonary blood flow, or to use the right ventricle and the proximal pulmonary artery to provide blood flow to the systemic circulation (18–20), with the pulmonary blood flow obtained with either a modified Blalock-Taussig shunt (21, 22) or with a right ventricle to pulmonary artery conduit (23).

The vast majority of children with balanced forms of univentricular heart require a surgical palliation early in life (4, 24–26). After the neonatal palliation, the Fontan circulation is generally established in two stages: an end-to-side connection between the SVC and the right pulmonary artery (bi-directional Glenn) (27–31), followed later by the Fontan completion, with the connection of the IVC to the pulmonary artery. Early techniques of direct atrio-pulmonary connection have been virtually abandoned in subsequent years, so that the two Fontan completion techniques most frequently utilized nowadays are the lateral tunnel, or intra-cardiac Fontan (32–34), and the extra-cardiac Fontan, connecting the transected stump of the IVC to the pulmonary artery with the interposition of a tubular prosthesis (35–38).

The long-term results of the Fontan circulation are complicated by substantial morbidity. This includes: chronic venous hypertension with increased hydrostatic capillary pressure, recurrent pericardial and pleural effusions, ascites, generalized fluid retention, renal failure, hepatic failure, gastro-intestinal dysfunction, supra-ventricular and ventricular arrhythmias, pulmonary and systemic thromboembolism, protein-losing enteropathy, pulmonary arterio-venous malformations and veno-venous collaterals, persistent and/or progressive hypoxemia, progressive impairment of the ventricular function, exercise intolerance, plastic bronchitis (39–69).

Mathematical models and computational fluid dynamic (CFD) studies have been extensively applied to assess the various types of cavo-pulmonary connections (70–87).

All current surgical techniques for the completion of the Fontan circulation deviate the blood from the SVC, which constitutes approximately 1/3 of the systemic venous return, to the right lung, occupying 60% of the total lung volume, while the blood from the IVC, which constitutes ~2/3 of the systemic venous return, is deviated to the left lung, with 40% of the total lung volume. Deviating the larger flow rate towards the lower volume lung and the converse suggests an unfavorable flow distribution, which may contribute to the poor long-term outcomes listed above.

The purpose of this study is to evaluate, using CFD models, a new “plumbing” for the completion of the Fontan circulation, with the SVC smaller venous return channeled toward the smaller left lung and the IVC larger venous return deviated toward the larger right lung, so that the ranking of the two blood flow rates matches the size ranking of the lungs. This study investigates whether this new proposal for the Fontan procedure, herein named “physiological” Fontan, can provide a better flow distribution than the “conventional” extra-cardiac Fontan.

## Materials and Methods

**Figure 1:**
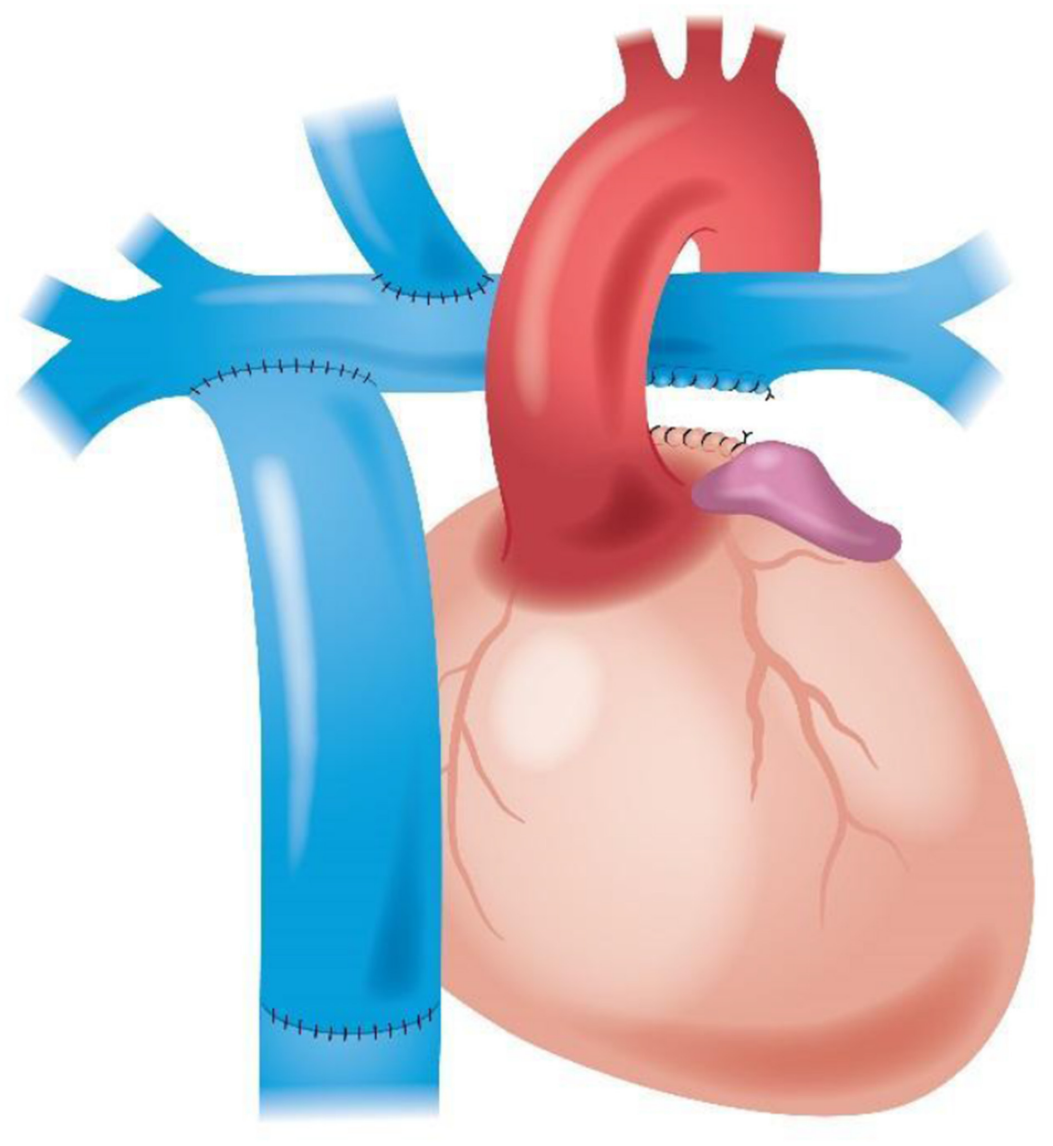
Conceptual arrangement of the new physiological Fontan procedure.

An *in-silico* three-dimensional (3D) parametric model of the physiological Fontan circulation was developed with the positions of the SVC and IVC connections varied along the pulmonary arteries (Figure 1). Idealized vascular geometries were constructed with constant dimensions of the SVC, the IVC, and the right and left pulmonary arteries. Steady velocity inflows for the IVC and SVC and constant equal outflow pressures for the right and left pulmonary arteries were set accordingly to established literature reports (70–84), to obtain finite-volume incompressible steady flow simulations, assuming a single-phase, Newtonian, isothermal, laminar blood flow.

The key reference data for dimensioning the vascular geometry were a 3.5 year old subject, body weight 16 kg, height 98 cm, body surface area 0.65 m^2^, and total cardiac output 2.6 L/min, with an indexed cardiac output of 4 L/min/m^2^. The pulmonary artery length ℓ, measured as the straight-line distance between the first branching of the right and left pulmonary arteries, was 7.3 cm. For comparison purposes, a baseline configuration was defined by modeling the conventional Fontan circulation, with the SVC confluence at 0.9 ℓ from the left pulmonary artery and the IVC confluence at 0.6 ℓ from the left pulmonary artery, with a right-angle (90°) connection for the SVC and a 75° connection facing toward the left pulmonary artery for the IVC. In the parametric model of the physiological Fontan, the IVC confluence was varied over the range 0.77 ℓ ≤ *x* ≤ 0.92 ℓ from the first branching of the left pulmonary artery. The IVC confluence angle with the pulmonary artery was varied with 15° increments between 60° and 90°. The SVC was connected to the pulmonary artery at a fixed angle of 60°. This connection was held at 0.50 ℓ from the first branching of the left pulmonary artery considering this as the maximum free length of transected SVC available after adequate surgical dissection when performing a bidirectional Glenn procedure.

These parametrized geometries defined the computational domain for finite-volume steady incompressible flow simulations, assuming a single-phase, Newtonian, isothermal, and laminar blood flow, of constant density ρ = 1060 kg m^−3^ and molecular viscosity 3.5 × 10^−3^ kg m^−1^ s^−1^. A 3D solid model of the computational domain was obtained using the commercial Computational Aided Design (CAD) package Solidworks 2018 (Dassaut Systèmes, Vélizy-Villacoublay, France), in which all surfaces were rendered as non-uniform rational basis splines (NURBs) for dimensional accuracy. Two mm radius fillets were applied at the edges of all vascular intersections to reproduce the natural behavior of the tissue that rounds off at the anastomoses.

**Figure 2:**
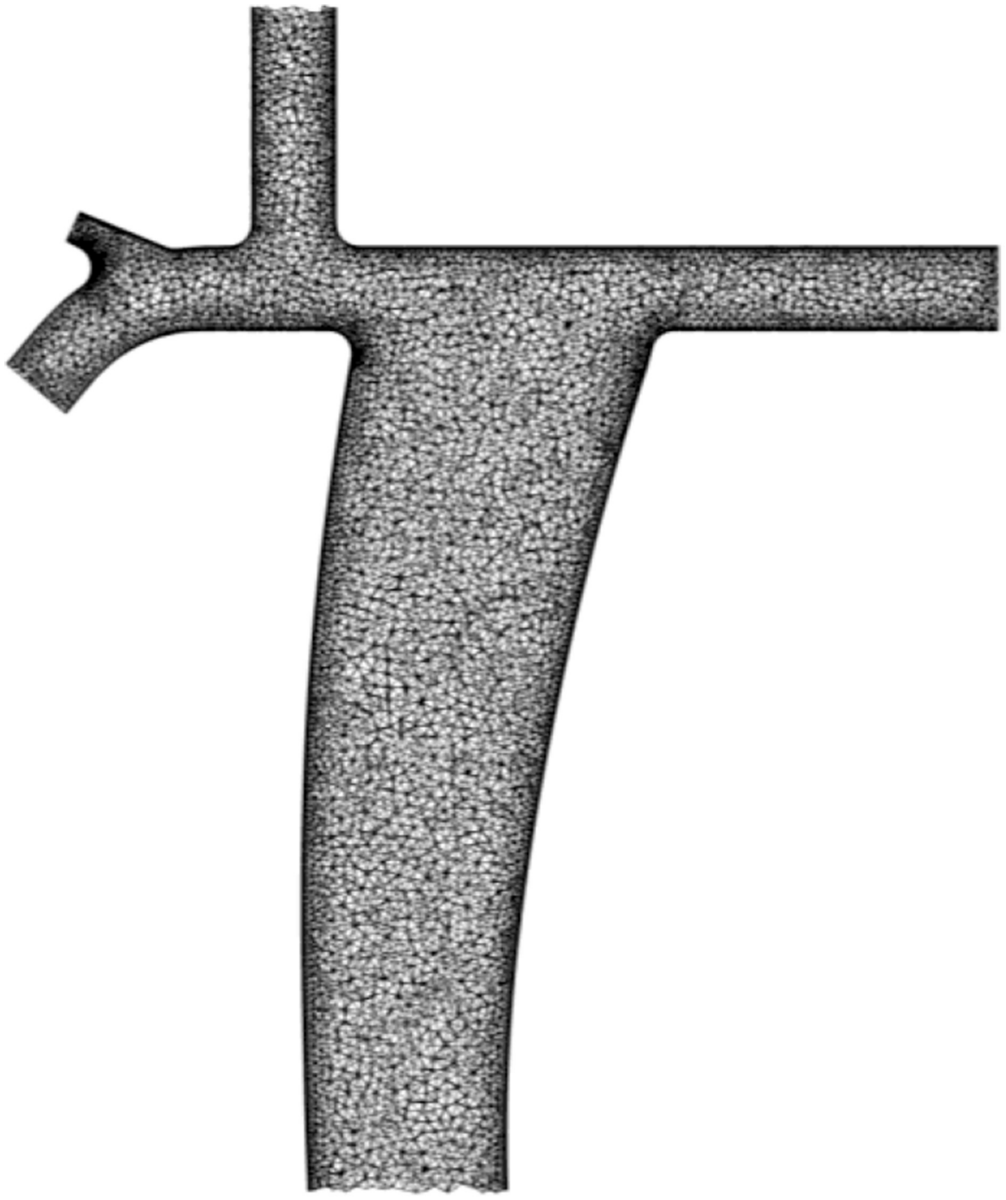
Computational domain of the conventional Fontan configuration meshed by a five-layers thick prism carpet mesh lining the walls that surrounds an unstructured tetrahedral mesh.

The computational domains were meshed by a five-layers thick prism carpet mesh lining the walls that surrounds an unstructured tetrahedral mesh. A sample mesh of a conventional Fontan configuration is shown in Figure 2. The spatial discretization was obtained by the commercial CFD pre-processor ICEM CFD by ANSYS Fluent (Ansys Inc., Canonsburg, Pennsylvania, USA). Care was taken to produce meshes with adequate cell orthogonality and aspect ratio in light of current CFD practice. The cell orthogonality and aspect ratio were used as mesh quality parameters. In the conventional Fontan, 1% of the cells have orthogonality <0.4 and 1% of the cells have aspect ratio higher than 11.1. These values were also representative of the meshes obtained for the parametrized geometry for the physiological Fontan. A judicious selection of the spatial discretization level was used, based on textbook examples of laminar flows in pipes (88). The appropriateness of this selection is tested later on in this article by determining the variability of the predictions over six different computational meshes, with 1M, 2M, 4M, 8M, 12M, and 18M cells, respectively, for both the conventional and the physiological Fontan settings.

Boundary conditions were imposed over all cells outwards-facing the computational domain. The no-slip condition was applied on all walls. Parabolic velocity profiles at SVC and IVC inlets were used with a prescribed steady mass flow rate. Specifically, the SVC drained 1/3 and the IVC carried 2/3 of the total systemic venous return. The right and left pulmonary arteries were set at constant equal outflow pressures as previously reported in other CFD studies (87). This fully defined the boundary value problem in hand.

Numerical solutions of the flow were obtained using the commercial CFD package ANSYS Fluent (Ansys Inc., Canonsburg, Pennsylvania, USA). Laminar flow simulations were obtained by application of the Semi-Implicit Method for Pressure Linked Equations (SIMPLE) (85), using a second order accurate upwind scheme for the velocity components (86). For numerical stability, under-relaxation factors of 0.7 and 0.4 were used for velocities and pressure, respectively. The convergence of the numerical solution was monitored by the residuals of the mass and of the momentum balances. Convergence was taken as a reduction to below 10^−4^ of the starting value of these residuals.

Numerically converged finite-volume mass and momentum flow balances determined the pressures and flow rates at the inlets and outlets from solving the model. Numerical closed-path integration of the energy fluxes across the domain boundaries determined the flow energy loss rate through the Fontan plumbing.

With invariant sizes of SVC (diameter = 7.5 mm), IVC (= 18 mm conduit), right (diameter = 7.5 mm) and left (diameter = 7.5 mm) pulmonary arteries, invariant flows from SVC and IVC (output indexed/body surface area = 4 L/min/m^2^ of body surface area), and invariant right and left pulmonary artery outflow pressures (= 12 mm Hg), the comparison between the conventional extra-cardiac Fontan and the physiological Fontan plumbing evaluated: (1) mean IVC pressure; (2) energy loss rate; (3) kinetic energy maximum value throughout the domain volume.

Twenty-one numerical experiments were conducted by systematically varying the IVC angle and location of confluence with the pulmonary artery (three angles and seven locations). The most promising configuration was identified based on the predicted lowest energy loss rate.

## Results

Energy loss rates for all the 21 tested configurations are reported in Table 1. Reported values are of the same order of magnitude as in previous CFD simulations of the Fontan circulation (75, 88, 89). The physiological Fontan with the IVC angle of confluence of 60° and centerline confluence *x* = 0.81 ℓ is the configuration that has comparatively the lowest energy loss rate among the 21 variants considered in this study. Hence this is singled out as the preferred configuration.

**Table 1:** Energy loss rate in mW from 21 configurations of “physiological” Fontan as predicted by CFD.

	**θ = 60°**	**θ = 75°**	**θ = 90°**
0.77 ℓ	5.66	5.94	6.02
0.78 ℓ	5.70	5.91	6.12
0.81 ℓ	5.55	5.80	5.79
0.85 ℓ	5.57	5.75	5.96
0.88 ℓ	5.71	5.91	6.08
0.91 ℓ	5.84	5.98	6.10
0.92 ℓ	5.88	6.01	6.19

**Figure 3:**
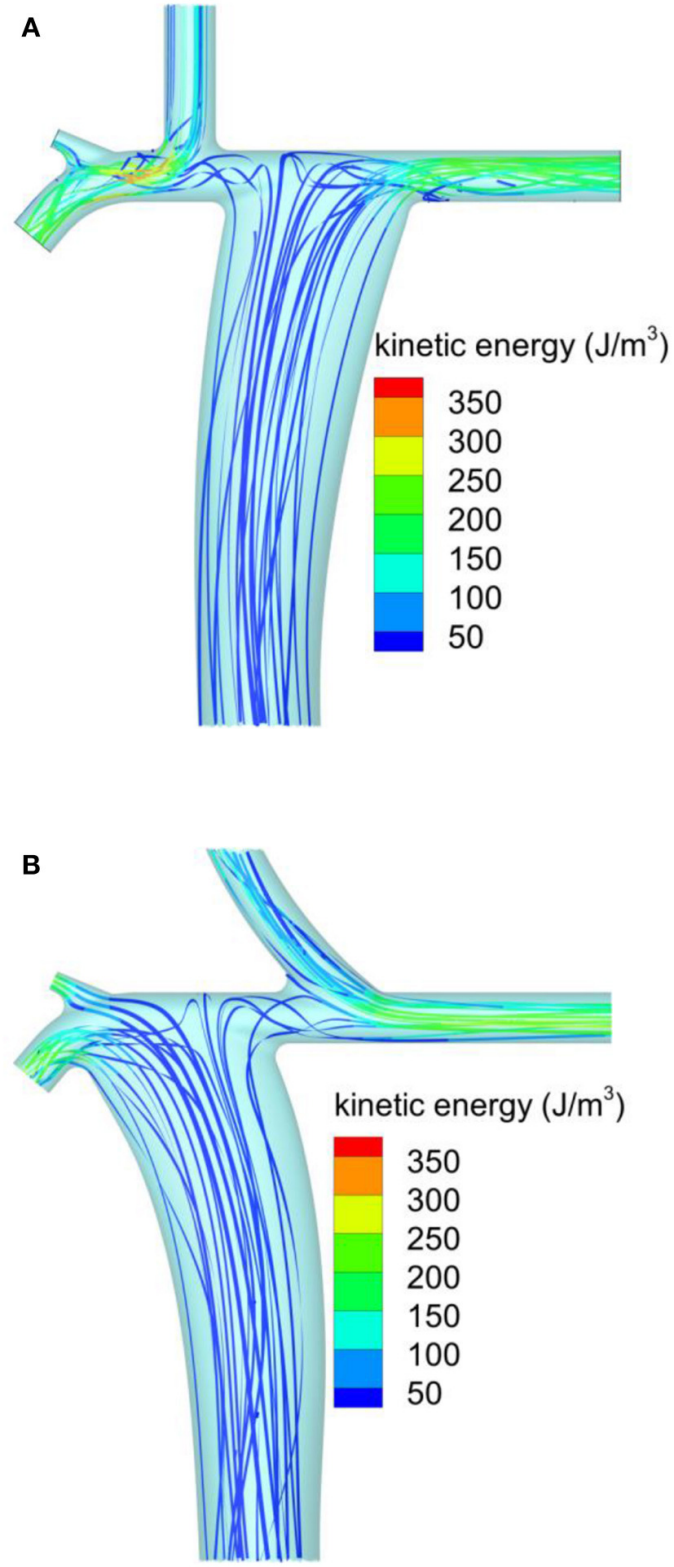
Streamlines predicted by CFD colored by the flow kinetic energy per unit volume: **(A)** conventional extracardiac Fontan; **(B)** new physiological Fontan.

A more detailed comparison between this configuration and the conventional Fontan was performed to identify flow features potentially responsible for the observed changes in the energy loss rate. Figure 3A shows the ribbons representing the predicted streamlines from the conventional Fontan model, using the 1M cells discretization. The ribbons were color coded by kinetic energy per unit volume. Most of the streamlines from the IVC were predicted to confluence toward the LPA, while the SVC was predicted to mainly supply the RPA (Figure 3A). The confluence from the SVC and IVC toward the RPA was characterized by localized flow acceleration that generated a kinetic energy peak shown in red in Figure 3A. This peak was about 350 J/m^3^. In the modeled laminar flow, energy was lost by viscous stresses caused by the velocity gradients in the flow. The kinetic energy peak indicated local high values of velocity and, by inference, a high velocity gradient to the surrounding stationary walls.

Figure 3B shows the corresponding predictions from the physiological Fontan, using the same notation and color scale for kinetic energy per unit volume as Figure 3A. Approximately the same spatial discretization of 1M cells was used. In the physiological Fontan, most of the streamlines from the IVC were predicted to confluence toward the RPA. The IVC confluence angle of 60° appeared to yield a less tortuous flow path through the PA compared to Figure 3A, as suggested by a reduced twisting in the ribbons just above the IVC anastomosis. In this configuration, the kinetic energy peak per unit volume was reduced to about 250 J/m^3^, as shown by the green iso-level, representing the peak iso-level in these ribbons. This reduced peak kinetic energy was expected to reduce the flow shear rate and thereby to reduce the viscous losses. This hypothesis was confirmed by the evaluation of the energy loss rate by integration over the inlets and outlets of the modeled vascular systems.

The comparison of the physiological vs. the conventional extra-cardiac Fontan provided the following results: (1) mean IVC pressure 13.9 vs. 14.1 mmHg (= 0.2 mmHg reduction); (2) energy loss rate 5.55 mW vs. 6.61 mW (= 16% reduction); (3) peak kinetic energy per unit volume 283 J/m^3^ vs. 396 J/m^3^ (= 29% reduction).

At this point, we tested the sensitivity of the predictions to the spatial discretization used in the model, which, although judiciously selected based on the established CFD practice (88), represented an arbitrary model input. To this end, the best performing physiological Fontan geometry was modeled on six different computational meshes of approximate node numbers of 1M, 2M, 4M, 8M, 12M, and 18M. Table 2 shows the predicted values from each computation, the ensemble mean, and the 95% confidence interval for the ensemble mean, based on the *t*-distribution of six samples. Table 3 shows the corresponding values from the conventional Fontan simulations.

**Table 2:** Performance of six realizations of the “physiological” Fontan, predicted on different CFD meshes.

	**Mean IVC pressure (mmHg)**	**Energy loss rate (mW)**	**Peak kinetic energy (J/m^**3**^)**
1M	13.92	5.55	284
2M	13.92	5.55	289
4M	13.95	5.66	290
8M	13.94	5.65	288
12M	13.96	5.67	292
18M	13.96	5.61	290
Mean	13.94 ± 0.017	5.62 ± 0.05	289 ± 3

**Table 3:** Performance of six realizations of the conventional Fontan, predicted on different CFD meshes.

	**Mean IVC pressure (mmHg)**	**Energy loss rate (mW)**	**Peak kinetic energy (J/m^**3**^)**
1M	14.05	6.61	396
2M	14.07	6.66	424
4M	14.05	6.54	421
8M	14.05	6.53	415
12M	14.05	6.53	423
18M	14.05	6.54	436
Mean	14.05 ± 0.008	6.57 ± 0.05	419 ± 13

Table 2 shows that the ensemble mean and 95% confidence interval for energy loss rate for the physiological Fontan is 5.62 ± 0.05 mW against the corresponding result for the conventional Fontan of 6.57 ± 0.05 mW from Table 3. There was no overlap between the confidence interval bands, therefore the two groups of simulations were separable. As a result, the reduction in energy loss rate was statistically significant, to a 95% confidence, as determined by the *t*-test. The physiological Fontan was confirmed as having a lower energy loss rate than the conventional Fontan.

The corresponding result for the mean IVC pressure is 13.94 ± 0.017 mmHg for the physiological Fontan against 14.05 ± 0.008 mmHg for the conventional Fontan. There was no overlap between the confidence interval bands, therefore the two groups of simulations were separable and showed that the mean IVC pressure from the physiological Fontan is numerically lower than that of the conventional Fontan. Specifically, the *t*-distribution suggests that, with a 95% confidence, the physiological Fontan is reducing the mean IVC pressure compared to the conventional Fontan. As these simulations used an idealized geometry, the magnitude of the change in IVC pressure has lower significance than the sign of this change, since the magnitude of this change is likely to differ from subject to subject.

Finally, Tables 2 and 3 report the maximum kinetic energy predicted by the two sets of simulations, as determined from the largest value of the scalar product of the velocity vectors in the computational domain interior. The kinetic energy values in Tables 2 and 3 are reported per unit volume, in the form and units of the flow dynamic pressure. This permitted to evaluate and locate the maximum of this intensive property in the modeled flow domain. The conventional Fontan is predicted to produce a flow with peak kinetic energy of 419 ± 13 J/m^3^, which is about one and a half the peak kinetic energy of 289 ± 3 J/m^3^ from the physiological Fontan. The *t*-distribution analysis indicates that the two sets of simulations (conventional and physiological Fontan) are also separable based on peak kinetic energy, as their mean difference is 130 J/m^3^ against a statistical uncertainty of 13.3 J/m^3^, to a 95% statistical confidence.

## Discussion

The idea of deviating the systemic venous return from the SVC toward the left lung and the systemic venous return from the IVC toward the right lung was already a matter of discussion between one of the authors (AFC) and Dr. Hillel Laks, cardiac surgeon at University of California, Los Angeles. Dr. Laks realized in a series of patients a modified connection of the SVC to the left pulmonary artery and of the IVC to the right pulmonary artery by completely dividing the right and the left pulmonary artery (90). This surgical technique obtained the distribution of the systemic venous returns to the appropriate size lungs, but unfortunately the separation of right and left pulmonary artery resulted in the left lung receiving only the blood from the SVC, therefore without the hepatic factor contained in the blood drained from the IVC. This combination is documented to cause pulmonary arterio-venous fistulas within a few years, with the subsequent reduction of oxygen saturation, with very poor clinical tolerance (39, 42, 44, 45, 49, 54, 55, 61).

The new physiological Fontan proposed in this study can obtain the appropriate blood flow distribution, matching the ranking of the systemic venous returns to the lung sizes ranking, without separating the right and left pulmonary arteries, and therefore allowing the blood from the IVC, containing the hepatic factor, to reach both lungs and hence prevent the complications of pulmonary arterio-venous fistulas.

The reduction in energy loss rate in the physiological Fontan was positively correlated to the reduction in the peak kinetic energy in the flow, which, as reported in Tables 2 and 3, was also separable and statistically significant. Flow visualization located the kinetic energy peak of the conventional Fontan at the confluence between the SVC and the IVC, toward the LPA. This peak is detrimental to the circulation effort through the vascular system, as kinetic energy is irreversibly lost by viscous stresses due to the velocity gradient between this fast moving flow and the stationary fluid wetting the walls of the Fontan vessels.

The reduction in energy loss rate associated to the physiological Fontan is enabled by the shallower confluence angles of the SVC and IVC with the PA, deviating these inflows respectively toward the LPA and RPA, thereby preserving the inflow momentum, compared to more right-angle confluences present in the conventional Fontan. In the clinical practice, the shallower angle confluence can be surgically accomplished only if the anastomoses of the SVC and IVC are transposed as in the physiological Fontan.

The physiological Fontan was also predicted, with 95% statistical confidence, to lower the IVC pressure compared to the conventional Fontan. The certainty in the direction of change of the IVC pressure is of prime importance, since high blood pressure has a strong causation link to liver and kidney failure in patients with Fontan circulation in the 20–30 years age group (43, 47, 50, 51, 60, 62, 64, 67, 69). The authors have used an idealized model for this comparative analysis, therefore the magnitude of the IVC pressure change is comparatively less significant, as this is dependent on the specific vascular geometry of the subject that is not rendered by the generalized geometry used in this work.

This surgical approach requires the first step of the Fontan circulation (bidirectional Glenn) with the end-to-side anastomosis of the superior vena cava obliquely moved toward the pulmonary artery bifurcation, instead that directly on the right pulmonary artery. This modification, deviating the flow toward the left lung, in principle should reduce the incidence of relative left pulmonary artery hypoplasia or small size frequently observed during the pre-operative investigations before Fontan completion, without interfering with the flow toward the right lung and therefore with the growth of the right pulmonary artery (91–93). By deviating the larger flow of the IVC towards the right lung, this new plumbing should also ensure that the right lung will not remain hypo-perfused after completion of the Fontan circulation.

As far as the pulmonary flow distribution is concerned, our results showed that, in addition to reducing the energy loss rate and the peak kinetic energy, the flow was uniformly divided between left and right pulmonary arteries in a very similar way as with the conventional Fontan.

### Limits of the Study

This preliminary CFD study aimed at addressing the fundamental question of whether a more “physiological” Fontan could provide a viable alternative to the “conventional” Fontan. Obtaining a general indication required adopting a very idealized geometry with uniform anatomy, obtained from 3D reconstruction of investigations in a real patient, that could express the essence of the difference between the vascular layouts under consideration. It would be beneficial to perform a CFD evaluation on subject-specific vascular systems, acquired by medical imaging, pre-operation, to obtain more specific predictions about this new surgical option. Pressure and flow rates acquired from clinical practice can inform the boundary conditions used in the CFD model, using an approach similar to the one described in previous reports (70–74, 80–87).

The steady flow modeling approach could be improved by repeating the simulations in a time-dependent CFD framework, using prescribed waveforms of mass flow rate at the SVC and IVC inflows (75) and adding a lumped mass parameter model of the dependence of the LPA and RPA outflow pressures on the flow rate through these outlets (75, 91). Here, the challenge is to obtain a lumped-parameter model that renders the difference in resistance and blood flow capacitance between the left and the right lungs (also including the effects of the respiratory cycle), which has so far not been modeled in numerical studies of Fontan circulation.

The CFD model assumed rigid and non-compliant walls, without the interferences with the walls caused by the flow, in line with a previous CFD study of the Fontan circulation (75). The significance of the variation of the lumen cross-section during the cardiac cycle should be assessed, as this may have a significant impact on temporal effects not rendered by the current steady flow CFD model.

Finally, whilst the proposed anastomosis of the SVC would be expected to reduce the incidence of hypoplasia of the left pulmonary artery, the perfusion of the left and of the right lungs after Glenn should be investigated numerically, to make sure the right lung is not significantly hypo-perfused before the Fontan completion, which may lead to the opposite problem of hypoplasia of the right pulmonary artery.

Despite all the above limits, mathematical and CFD studies are still justified as methods for the first step in the “*in vitro*” evaluation of the cavopulmonary connections, even though currently they have to deal with all the variables present in the biological environment. Animals with univentricular hearts (such as frogs and turtles), are not suitable for experimental studies of cavopulmonary connections, and all animal models used to perform hemodynamic evaluation of cavopulmonary connections only allowed acute studies, as in our previous experience (66) and as confirmed by a recent systematic review (94).

## Conclusions

This *in silico* study confirmed that the proposed Fontan configuration characterized by a more physiological flow distribution is accompanied by a little reduction of mean IVC pressure and by a substantial reduction of energy loss rate and of peak kinetic energy. The potential clinical impact of these hemodynamic changes is to improve the long-term outcomes of the Fontan circulation, in particular to reduce the adverse incidence of liver failure and protein-losing enteropathy. Further studies with *in vitro* mock circuits should provide more information about the potential advantages on fluid dynamics of the new physiological Fontan procedure. In particular, they could provide more information about the magnitude of the IVC pressure reduction that can be achieved by the physiological Fontan compared to the conventional Fontan, by regressing flow measurements obtained from different vascular geometries based on subject-specific measurements of the vascular system.

## Data Availability

The raw data supporting the conclusions of this manuscript will be made available by the authors, without undue reservation, to any qualified researcher.

## Author Contributions

AFC proposed the new procedure, collaborated to the preparation of the manuscript and the final revision. MO, AC, and EH produced the mathematical and CFD models used for the evaluations in the study, running all the required computations, and reviewed the manuscript. AR contributed and supervised the mathematical and CFD evaluations of the study, combining the requirements of the mathematical and clinical aspects, contributed to the preparation of the manuscript, and revised the final edition.

### Conflict of Interest Statement

The authors declare that the research was conducted in the absence of any commercial or financial relationships that could be construed as a potential conflict of interest.

## References

[1] Fontan F Baudet E. Surgical repair of tricuspid atresia. Thorax. (1971) 26:240–8. 10.1136/thx.26.3.240 5089489 PMC1019078

[2] Anderson RH Becker AE Wilkinson JL. Morphogenesis and nomenclature of univentricular hearts. Br Heart J. (1975) 37:781. 1156496

[3] Marcelletti C Mazzera E Olthof H Sebel PS Duren DR Losekoot TG. Fontan's operation: an expanded horizon. J Thorac Cardiovasc Surg. (1980) 80:764. 7431973

[4] Corno AF Becker AE Bulterijs AHK Lam J Nijveld A Schuller C. Univentricular heart: can we alter the natural history? Ann Thorac Surg. (1982) 34:716–26. 10.1016/S0003-4975(10)60917-4 6184025

[5] Fontan F Kirklin JW Fernandez G Costa F Naftel DC Tritto F. Outcome after a “perfect” Fontan operation. Circulation. (1990) 81:1520–36. 10.1161/01.CIR.81.5.1520 2331765

[6] Amodeo A Galletti L Marianeschi S Picardo S Giannico S di Renzi P. Extracardiac Fontan operation for complex cardiac anomalies: seven years' experience. J Thorac Cardiovasc Surg. (1997) 114:1020–30. 10.1016/S0022-5223(97)70016-3 9434697

[7] de Leval MR. The Fontan circulation: what have we learned? What to expect? Pediatr Cardiol. (1998) 19:316–20. 10.1007/s002469900315 9636255

[8] Gewillig M. The Fontan circulation. Heart. (2005) 91:839–46. 10.1136/hrt.2004.051789 15894794 PMC1768934

[9] Blalock A Taussig HB The surgical treatment of malformations of the heart in which there is pulmonary stenosis or pulmonary atresia. JAMA. (1945) 128:189–92. 10.1001/jama.1945.02860200029009 6368878

[10] De Leval MR McKay R Jones M Stark J Macartney FJ. Modified Blalock-Taussig shunt. Use of subclavian artery orifice as flow regulator in prosthetic systemic-pulmonary shunts. J Thorac Cardiovasc Surg. (1981) 81:112. 6450303

[11] Corno AF Hurni M Tozzi P von Segesser LK. Accordion-like prosthesis for modified Blalock-Taussig shunt. Asian Cardiovasc Thorac Ann. (2003) 11:229–32. 10.1177/021849230301100311 14514554

[12] Muller WH Dammann JF. The treatment of certain congenital malformations of the heart by the creation of pulmonic stenosis to reduce pulmonary hypertension and excessive pulmonary blood flow: a preliminary report. Surg Gynecol Obstet. (1952) 95:213. 14950654

[13] Trusler GA Mustard WT. A method of banding the pulmonary artery for large isolated ventricular septal defect with and without transposition of the great arteries. Ann Thorac Surg. (1972) 13:351. 10.1016/S0003-4975(10)64866-7 5019859

[14] Freedom RM Sondheimer H Dische R Rowe RD. Development of “subaortic stenosis” after pulmonary arterial banding for common ventricle. Am J Cardiol. (1977) 39:78–83. 10.1016/S0002-9149(77)80015-5 556661

[15] Freedom RM. The dinosaur and banding of the main pulmonary trunk in the heart with functionally one ventricle and transposition of the great arteries: a saga of evolution and caution. J Am Coll Cardiol. (1987) 10:427–9. 10.1016/S0735-1097(87)80028-1 2955026

[16] Corno AF Bonnet D Sekarski N Sidi D Vouhé PR von Segesser LK. Remote control of pulmonary blood flow: initial clinical experience. J Thorac Cardiovasc Surg. (2003) 126:1775–80. 10.1016/j.jtcvs.2003.06.011 14688686

[17] Corno AF Ladusans EJ Pozzi M Kerr S FloWatch® versus conventional pulmonary artery banding. J Thorac Cardiovasc Surg. (2007) 134:1413–9. 10.1016/j.jtcvs.2007.03.065 18023654

[18] Lin AE Laks H Barber G Chin AJ Williams RG. Subaortic obstruction in complex congenital heart disease: management by proximal pulmonary artery to ascending aorta end-to-side anastomosis. J Am Coll Cardiol. (1986) 7:617–24. 10.1016/S0735-1097(86)80473-9 3950241

[19] Karl TR Watterson KG Sano S Mee RBB. Operations for subaortic stenosis in univentricular hearts. Ann Thorac Surg. (1991) 52:420. 10.1016/0003-4975(91)90901-2 1898129

[20] Gates RN Laks H Elami A Drinkwater DC Pearl JM George BL. Damus-Stansel-Kaye procedure: current indications and results. Ann Thorac Surg. (1993) 56:111–9. 10.1016/0003-4975(93)90413-C 8328840

[21] Norwood WI Lang P Castaneda AR Campbell DN. Experience with operations for hypoplastic left heart syndrome. J Thorac Cardiovasc Surg. (1981) 82:511–9. 6168869

[22] Norwood WI Lang P Hansen DD. Physiologic repair of aortic atresia-hypoplastic left heart syndrome. N Engl J Med. (1983) 308:23–6. 10.1056/NEJM198301063080106 6847920

[23] Sano S Ishino K Kawada M Fujisawa E Kasahara S Nakanishi K The modified Norwood operation for hypoplastic left heart syndrome using right ventricle-to-pulmonary artery shunt. Cardiol Young. (2004) 14(Suppl 3):90–5.15903112

[24] Somerville J. Changing form and function in one ventricle hearts. Herz. (1979) 4:206–12. 571834

[25] Moodie DS Ritter DG Tajik AH O'Fallon WM. Long-term follow-up in the unoperated univentricular heart. Am J Cardiol. (1984) 53:1124–8. 10.1016/0002-9149(84)90648-9 6702691

[26] Hager A Kaemmerer H Eicken A Fratz S Hess J Long-term survival of patients with univentricular heart not treated surgically. J Thorac Cardiovasc Surg. (2002) 123:1214–7. 10.1067/mtc.2002.122535 12063473

[27] Corno AF Mazzera E Marino B Picardo S Marcelletti C Bidirectional cavopulmonary anastomosis. J Am Coll Cardiol. (1989) 13:74A. 10.1016/0003-4975(89)90384-6 2467631

[28] Mazzera E Corno AF Picardo S Di Donato RM Marino B Costa D Bidirectional cavopulmonary shunts: clinical applications as staged or definitive palliation. Ann Thorac Surg. (1989) 47:415–20. 10.1016/0003-4975(89)90384-6 2467631

[29] Bridges ND Jonas RA Mayer JE Flanagan MF Keane JF Castaneda AR. Bidirectional cavopulmonary anastomosis as interim palliation for high risk Fontan candidates. Circulation. (1990) 82(suppl. IV):170–6. 1699686

[30] Freedom RM Nykanen D Benson LN. The physiology of the bidirectional cavopulmonary connection. Ann Thorac Surg. (1998) 66:664–7. 10.1016/S0003-4975(98)00618-3 9725449

[31] Eyskens B Mertens L Kuzo R De Jaegere T Lawrenson J Dymarkowski S The ratio of flow in the superior and inferior caval veins after construction of a bi-directional cavopulmonary anastomosis in children. Cardiol Young. (2003) 13:123–30. 10.1017/S1047951103000258 12887067

[32] de Leval MR Kilner P Gewillig M Bull C. Total cavopulmonary connection: a logical alternative to atriopulmonary connection for complex Fontan operations. J Thorac Cardiovasc Surg. (1988) 96:682–95. 3184963

[33] Jonas RA Castaneda AR Modified Fontan procedure: atrial baffle and systemic venous to pulmonary artery anastomotic technique. J Card Surg. (1998) 3:91–6. 10.1111/j.1540-8191.1988.tb00228.x 2980014

[34] Stamm C Friehs I Mayer JE Zurakowski D Triedman JK Moran AM. Long-term results of the lateral tunnel Fontan operation. J Thorac Cardiovasc Surg. (2001) 121:28–41. 10.1067/mtc.2001.111422 11135157

[35] Marcelletti C Corno AF Giannico S Marino B. Inferior vena cava to pulmonary artery extracardiac conduit: a new form of right heart bypass. J Thorac Cardiovasc Surg. (1990) 100:228–232. 2143549

[36] Giannico S Corno AF Marino B Cicini MP Gagliardi MG Amodeo A. Total extracardiac right heart bypass. Circulation. (1992) 86(suppl. II):110–7. 1423988

[37] Burke RP Jacobs JP Ashraf MH Aldousany A Chang AC. Extracardiac Fontan operation without cardiopulmonary bypass. Ann Thorac Surg. (1997) 63:1175–7. 10.1016/S0003-4975(97)00191-4 9124936

[38] Petrossian E Reddy VM McElhinney DB Akkersdijk GP Moore P Parry AJ. Early results of the extracardiac conduit Fontan operation. J Thorac Cardiovasc Surg. (1999) 117:688–96. 10.1016/S0022-5223(99)70288-6 10096963

[39] Cloutier A Ash JM Smallhorn JF Williams WG Trusler GA Rowe RD. Abnormal distribution of pulmonary blood after the Glenn shunt or Fontan procedure: risk of development of arteriovenous fistulae. Circulation. (1985) 72:471–9. 10.1161/01.CIR.72.3.471 4017202

[40] Ilbawi MN Idriss FS Muster AJ DeLeon SY Berry TE Duffy E. Effects of elevated coronary sinus pressure on left ventricular function after the Fontan operation. J Thorac Cardiovasc Surg. (1986) 92:231. 3736081

[41] Cecchin F Johnsrude CL Perry JC Friedman RA Effect of age and surgical technique on symptomatic arrhythmias after the Fontan operation. Am J Cardiol. (1995) 76:386–91. 10.1016/S0002-9149(99)80106-4 7639165

[42] Srivastava D Preminger T Lock JE Mandell V Keane JF Mayer JE. Hepatic venous blood and the development of pulmonary arteriovenous malformations in congenital heart disease. Circulation. (1995) 92:1217–22. 10.1161/01.CIR.92.5.1217 7648668

[43] Gentles TL Gauvreau K Mayer JE Fishberger SB Burnett J Colan SD. Functional outcome after the Fontan operation: factors influencing late morbidity. J Thorac Cardiovasc Surg. (1997) 114:392–403. 10.1016/S0022-5223(97)70184-3 9305191

[44] Marshall JB Duncan BW Jonas AR The role of angiogenesis in the development of pulmonary arteriovenous malformations in children after cavopulmonary anastomosis. Cardiol Young. (1997) 7:370–4. 10.1017/S1047951100004352

[45] Shah MJ Rychik J Fogel MA Murphy JD Jacobs ML Pulmonary arteriovenous malformations after superior cavopulmonary connection: resolution after inclusion of hepatic veins in the pulmonary circulation. Ann Thorac Surg. (1997) 63:960–3. 10.1016/S0003-4975(96)00961-7 9124971

[46] Durongpisitkul K Porter CJ Cetta F Offord KP Slezak JM Puga FJ. Predictors of early- and late-onset supraventricular tachyarrhythmias after Fontan operation. Circulation. (1998) 98:1099–107. 10.1161/01.CIR.98.11.1099 9736597

[47] Mertens L Hagler DJ Sauer U Somerville J Gewillig M. Protein-losing enteropathy after the Fontan operation: an international multicenter study. J Thorac Cardiovasc Surg. (1998) 115:1063–73. 10.1016/S0022-5223(98)70406-4 9605076

[48] Troutman WB Barstow TJ Galindo AJ Cooper DM. Abnormal dynamic cardiorespiratory responses to exercise in pediatric patients after Fontan procedure. J Am Coll Cardiol. (1998) 31:668–73. 10.1016/S0735-1097(97)00545-7 9502651

[49] Chang RK Alejos JC Atkinson D Jensen R Drant S Galindo A. Bubble contrast echocardiography in detecting pulmonary arteriovenous shunting in children with univentricular heart after cavopulmonary anastomosis. J Am Coll Cardiol. (1999) 33:2052–8. 10.1016/S0735-1097(99)00096-0 10362213

[50] Jahangiri M Kreutzer J Zurakowski D Bacha E Jonas RA Evaluation of hemostasis and coagulation factor abnormalities in patients undergoing the Fontan operation. J Thorac Cardiovasc Surg. (2000) 120:778–82. 10.1067/mtc.2000.108903 11003762

[51] Buchhorn R Bartmus D Buhre W Bursch J. Pathogenetic mechanisms of venous congestion after the Fontan procedure. Cardiol Young. (2001) 11:161–8. 10.1017/S1047951101000051 11293733

[52] Cohen MI Rhodes LA Wernovsky G Gaynor JW Spray TL Rychik J. Atrial pacing: an alternative treatment for protein-losing enteropathy after the Fontan operation. J Thorac Cardiovasc Surg. (2001) 121:582–3. 10.1067/mtc.2001.110681 11241095

[53] Coon PD Rychik J Novello RT Rao PS Gaynor JW Spray TL. Thrombus formation after the Fontan operation. Ann Thorac Surg. (2001) 71:1990–4. 10.1016/S0003-4975(01)02472-9 11426780

[54] Heinemann M Breuer J Steger V Steil E Sieverding L Ziemer G. Incidence and impact of systemic venous collateral development after Glenn and Fontan procedures. Thorac Cardiovasc Surg. (2001) 49:172–8. 10.1055/s-2001-14339 11432477

[55] Ashrafian H Swan L. The mechanism of formation of pulmonary arteriovenous malformations associated with the classic Glenn shunt (superior cavopulmonary anastomosis). Heart. (2002) 88:639. 10.1136/heart.88.6.639 12433901 PMC1767473

[56] Mavroudis C Deal BJ Backer CL. The beneficial effects of total cavopulmonary conversion and arrhythmia surgery for the failed Fontan. Semin Thorac Cardiovasc Surg Pediatr Card Surg Ann. (2002) 5:12–24. 10.1053/pcsu.2002.31489 11994861

[57] Monagle P Karl TR. Thromboembolic problems after the Fontan operation. Semin Thorac Cardiovasc Surg Pediatr Card Surg Ann. (2002) 5:36–47. 10.1053/pcsu.2002.29716 11994863

[58] Piran S Veldtman G Siu S Webb GD Liu PP Heart failure and ventricular dysfunction in patients with single or systemic right ventricles. Circulation. (2002) 105:1189–94. 10.1161/hc1002.105182 11889012

[59] Deal BJ Mavroudis C Backer CL. Beyond Fontan conversion: surgical therapy of arrhythmias including patients with associated complex congenital heart disease. Ann Thorac Surg. (2003) 76:542–54. 10.1016/S0003-4975(03)00469-7 12902101

[60] Dearani JA Danielson GK Puga FJ Schaff HV Warnes CW Driscoll DJ. Late follow-up of 1095 patients undergoing operation for complex congenital heart disease utilizing pulmonary ventricle to pulmonary artery conduits. Ann Thorac Surg. (2003) 75:399–411. 10.1016/S0003-4975(02)04547-2 12607647

[61] Duncan BW Desai S Pulmonary arteriovenous malformations after cavopulmonary anastomosis. Ann Thorac Surg. (2003) 276:1759–66. 10.1016/S0003-4975(03)00450-8 14602341

[62] Narkewicz MR Sondheimer HM Ziegler JW Hepatic dysfunction following the Fontan operation. J Pediatr Gastroenterol Nutr. (2003) 36:352–7. 10.1097/00005176-200303000-00009 12604973

[63] Odegard KC McGowan FX Zurakowski D DiNardo JA Castro RA del Nido PJ. Procoagulant and anticoagulant factor abnormalities following the Fontan procedure: increased factor VIII may predispose to thrombosis. J Thorac Cardiovasc Surg. (2003) 125:1260–7. 10.1016/S0022-5223(02)73605-2 12830042

[64] Giannico S Hammad F Amodeo A Michielon G Drago F Turchetta A. Clinical outcome of 193 extracardiac Fontan patients: the first 15 years. J Am Coll Cardiol. (2006) 47:2065–73. 10.1016/j.jacc.2005.12.065 16697327

[65] de Leval MR Deanfield JE. Four decades of Fontan palliation. Nat Rev Cardiol. (2010) 7:520–7. 10.1038/nrcardio.2010.99 20585329

[66] Corno AF Vergara C Subramanian C Johnson RA Passerini T Veneziani A. Assisted Fontan procedure: animal and *in vitro* models and computational fluid dynamics study. Interact CardioVasc Thorac Surg. (2010) 10:679–84. 10.1510/icvts.2009.223024 20123892

[67] Pundi KN Johnson JN Dearani JA Pundi KN Li Z Hinck CA. 40-year follow-up after the Fontan operation: long-term outcomes of 1,052 patients. J Am Coll Cardiol. (2015) 66:1700–10. 10.1016/j.jacc.2015.07.065 26449141

[68] Pundi KN Pundi KN Johnson JN Dearani JA Li Z Driscoll DJ. Sudden cardiac death and late arrhythmias after the Fontan operation. Congenit Heart Dis. (2017) 12:17–23. 10.1111/chd.12401 27545004

[69] Dennis M Zannino D du Plessis K Bullock A Disney PJS Radford DJ. Clinical outcomes in adolescents and adults after the Fontan procedure. J Am Coll Cardiol. (2018) 71:1009–17. 10.1016/j.jacc.2017.12.054 29495980

[70] Van Haesdonck JM Mertens L Sizaire R Montas G Purnode B Daenen W. Comparison by computerized numeric modeling of energy losses in different Fontan connections. Circulation. (1995) 92(suppl. II):322–6. 10.1161/01.CIR.92.9.322 7586432

[71] Laks H Ardehali A Grant PW Permut L Aharon A Kuhn M Computer simulation of circulation in patient with total cavo-pulmonary connection: inter-relationship of cardiac and vascular pressure, flow, resistance and capacitance. Med Biol Eng Comput. (1997) 35:722–8. 10.1007/BF02510984 9538552

[72] Ascuitto RJ Kydon DW Ross-Ascuitto NT. Pressure loss from flow energy dissipation: relevance to Fontan-type modifications. Pediatr Cardiol. (2001) 22:110–5. 10.1007/s002460010172 11178663

[73] Sharma S Ensley AE Hopkins K Chatzimavroudis GP Healy TM Tam VK. *In vivo* flow dynamics of the total cavopulmonary connection from three-dimensional multislice magnetic resonance imaging. Ann Thorac Surg. (2001) 71:889–98. 10.1016/S0003-4975(00)02517-0 11269470

[74] Bove EL de Leval MR Migliavacca F Guadagni G Dubini G. Computational fluid dynamics in the evaluation of hemodynamic performance of cavopulmonary connections after the Norwood procedure for hypoplastic left heart syndrome. J Thorac Cardiovasc Surg. (2003) 126:1040–7. 10.1016/S0022-5223(03)00698-6 14566244

[75] Migliavacca F Dubini G Bove EL de Leval MR. Computational fluid dynamics simulations in realistic 3-D geometries of the total cavopulmonary anastomosis: the influence of the inferior caval anastomosis. J Biomech Engineer. (2003) 125:805–13. 10.1115/1.1632523 14986405

[76] de Leval MR The Fontan circulation: a challenge to William Harvey? Nat Clin Pract Cardiovasc Med. (2005) 4:202–8. 10.1038/ncpcardio0157 16265484

[77] Itatani K Miyaji K Tomoyasu T Nakahata Y Ohara T Takamoto S. Optimal conduit size of the extracardiac Fontan operation based on energy loss and flow stagnation. Ann Thorac Surg. (2009) 88:565–73. 10.1016/j.athoracsur.2009.04.109 19632413

[78] Itatani K Miyaji K Nakahata Y Ohara K Takamoto S Ishii M. The lower limit of the pulmonary artery index for the extracardiac Fontan circulation. J Thorac Cardiovasc Surg. (2011) 142:127–35. 10.1016/j.jtcvs.2010.11.033 21277599

[79] Mirabella L Haggerty CM Passerini T Piccinelli M Powell AJ del Nido PJ. Treatment planning for a TCPC test case: a numerical investigation under rigid and moving wall assumptions. Int J Numer Method Biomed Eng. (2013) 29:197–216. 10.1002/cnm.2517 23345252

[80] Restrepo M Luffel M Sebring J Kanter KR del Nido PJ Veneziani A. Surgical planning of the total cavopulmonary connection: Robustness analysis. Ann Biomed Eng. (2015) 43:1321–34. 10.1007/s10439-014-1149-7 25316591 PMC4398591

[81] Trusty PM Restrepo M Kanter KR Yoganathan AP Fogel MA Slesnick TC. A pulsatile hemodynamic evaluation of the commercially available bifurcated Y-graft Fontan modification and comparison with the lateral tunnel and extracardiac conduits. J Thorac Cardiovasc Surg. (2016) 151:1529–36. 10.1016/j.jtcvs.2016.03.019 27056758

[82] Restrepo M Colleen Crouch A Haggerty CM Rossignac J Slesnick TC Kanter KR. Hemodynamic impact of superior vena cava placement in the Y-graft Fontan connection. Ann Thorac Surg. (2016) 101:183–9. 10.1016/j.athoracsur.2015.07.012 26431925

[83] Wei ZA Trusty PM Tree P Haggerty CM Tang E Fogel M. Can time-averaged flow boundary conditions be used to meet the clinical timeline for Fontan surgical planning? J Biomech. (2017) 50:172–9. 10.1016/j.jbiomech.2016.11.025 27855985 PMC5191925

[84] Trusty PM Wei Z Rychik J Russo PA Surrey LF Goldberg DJ. Impact of hemodynamics and fluid energetics on liver fibrosis after Fontan operation. J Thorac Cardiovasc Surg. (2018) 156:267–75. 10.1016/j.jtcvs.2018.02.078 29609888

[85] Patankar SV Spalding DB A calculation procedure for heat, mass and momentum transfer in three-dimensional parabolic flows. Int J Heat Mass Transfer. (1972) 15:1787–806. 10.1016/0017-9310(72)90054-3

[86] van Leer B Towards the ultimate conservative difference scheme, V. A second order sequel to Godunov's method. J Com Phys. (1979) 32:101–36. 10.1016/0021-9991(79)90145-1

[87] DeGroff CG. Modeling the Fontan Circulation: where we are and where we need to go. Pediatr Cardiol. (2008) 29:3–12. 10.1007/s00246-007-9104-0 17917765

[88] Anderson JD Jr Computational Fluid Dynamics, The Basics With Applications. New York, NY: McGraw-Hill Inc (1995).

[89] Ding J Liu Y Wang F. Influence of bypass angles on extracardiac Fontan connections: a numerical study. Int J Numer Method Biomed Eng. (2013) 29:351–62. 10.1002/cnm.2508 23345174

[90] Laks H Ardehali A Grant PW Permut L Aharon A Kuhn M. Modification of the Fontan procedure. Superior vena cava to left pulmonary artery connection and inferior vena cava to right pulmonary artery connection with adjustable atrial septal defect. Circulation. (1995) 91:2943–7. 10.1161/01.CIR.91.12.2943 7796504

[91] Hong H Menon PG Zhang H Ye L Zhu Z Chen H. Postsurgical comparison of pulsatile hemodynamics in five unique total cavopulmonary connections: identifying ideal connection strategies. Ann Thorac Surg. (2013) 96:1398–405. 10.1016/j.athoracsur.2013.05.035 23910632

[92] Hosein RB Clarke AJ McGuirk SP Griselli M Stumper O De Giovanni JV Factors influencing early and late outcome following the Fontan procedure in the current era. The “Two Commandments”? Eur J Cardiothorac Surg. (2007) 31:344–52. 10.1016/j.ejcts.2006.11.043 17236782

[93] Nassar MS Bertaud S Goreczny S Greil G Austin CB Salih C Technical and anatomic factors affecting the size of the branch pulmonary arteries following first-stage Norwood palliation for hypoplastic left heart syndrome. Interact Cardiovasc Thorac Surg. (2015) 20:631–5. 10.1093/icvts/ivv002 25681509

[94] Granegger M Valencia A Quandt D Dave H Kretschmar O Hübler M. Approaches to establish extracardiac total cavopulmonary connections in animal models; a review. World J Ped Congenit Heart Surg. (2019) 10:81–9. 10.1177/2150135118802788 30799726

